# Delayed diagnosis of complex glycerol kinase deficiency in a Chinese male infant: a case report

**DOI:** 10.1186/s12887-022-03568-9

**Published:** 2022-09-01

**Authors:** Na Tao, Xiaomei Liu, Yueqi Chen, Meiyuan Sun, Fang Xu, Yanfang Su

**Affiliations:** 1 Department of Endocrinology and Metabolism, Kunming Children’s Hospital / Affiliated Children’s Hospital of Kunming Medical University, No. 288 Qianxing Road, Xishan district, 650228 Kunming, China; 2 Kunming Key Laboratory of Children Infection and Immunity, Kunming Children’s Hospital, Kunming, China; 3 Yunnan Key Laboratory of Children’s Major Disease Research, Kunming Children’s Hospital, Kunming, China; 4 Department of Endocrinology, Yunnan Provincial Hospital of Traditional Chinese Medicine, Kunming, China

**Keywords:** Complex glycerol kinase deficiency, Infant, Xp21 contiguous gene deletion syndrome, Corticosteroid replacement

## Abstract

**Background:**

Xp21 contiguous gene deletion syndrome is a rare genetic metabolic disorder with poor prognosis in infants, involving deletions of one or more genes in Xp21. When deletions of adrenal hypoplasia (AHC), Duchenne muscular dystrophy (DMD), and chronic granulomatosis (CGD) loci are included, complex glycerol kinase deficiency (CGKD) can be diagnosed. We present a case of CGKD that was initially misdiagnosed and died during treatment in our hospital in terms of improving our understanding of the clinical features and diagnosis of this disease, as well as highlighting the need for more precise dosing of corticosteroid replacement therapy.

**Case presentation:**

A 48-day-old full-term male infant was transferred to our medical center with global growth delay and persistent vomiting. Routine laboratory tests revealed hyperkalemia, hyponatremia, and a high level of creatine kinase. The initial diagnosis was adrenal cortical hyperplasia (ACH), then revised to adrenocortical insufficiency with a normal level of ACTH detected. After supplementing the routine lipid test and urinary glycerol test, CGKD was diagnosed clinically due to positive triglyceridemia and urinary glycerol, and the follow-up gene screening further confirmed the diagnosis. The boy kept thriving after corticosteroid replacement and salt supplementation. While levels of serum ACTH and cortisol decreased and remained low after corticosteroid replacement was administered. The patient died of acute type 2 respiratory failure and hypoglycemia after an acute upper respiratory tract infection, which may be the result of adrenal crisis after infection. Infants with CGKD have a poor prognosis, so physicians should administer regular follow-ups, and parents counseling during treatment to improve the survival of patients.

**Conclusions:**

Overall, CGKD, although rare, cannot be easily excluded in children with persistent vomiting. Extensive blood tests can help to detect abnormal indicators. Adrenal crisis needs to be avoided as much as possible during corticosteroid replacement therapy.

## Introduction

Xp21 contiguous gene deletion syndrome, a rare genetic metabolic disorder, is the result of the deletion of a chromosomal fragment in the Xp21 region that contains the glycerol kinase (GK) locus [1]. The loci for adrenal hypoplasia (AHC), Duchenne muscular dystrophy (DMD), chronic granulomatosis (CGD), ornithine carbamoyltransferase (OTC) deficiency, and retinitis pigmentosa (RP) are commonly involved. The AHC and DMD loci are the closest to the glycerol kinase deficiency (GKD) locus, making the combined AHC-GKD-DMD the most common genotype of this syndrome known as Complex glycerol kinase deficiency (CGKD) [2, 3].

According to previous reports, this syndrome can be sporadic or familial, and genotypes do not predict phenotypes [1, 4]. This syndrome is more common in boys [5]. The variability of the affected gene fragments leads to multiple phenotypes. Symptoms can appear in infancy or childhood, with growth delay or mental retardation as the main symptoms [3]. The variability of clinical symptoms of CGKD also makes diagnosis difficult [6] and infants with CGKD may die in an early life without proper management.

We report an initial misdiagnosed case of Xp21 contiguous gene deletion syndrome in our hospital. This case involved a male infant with a delayed diagnosis of GKD and died of respiratory failure and hypoglycemia after an acute upper respiratory tract infection. The case also supports an understanding of the clinical features, pathogenesis, treatment, and prognosis of the disease.

## Case presentation

**Fig. 1:**
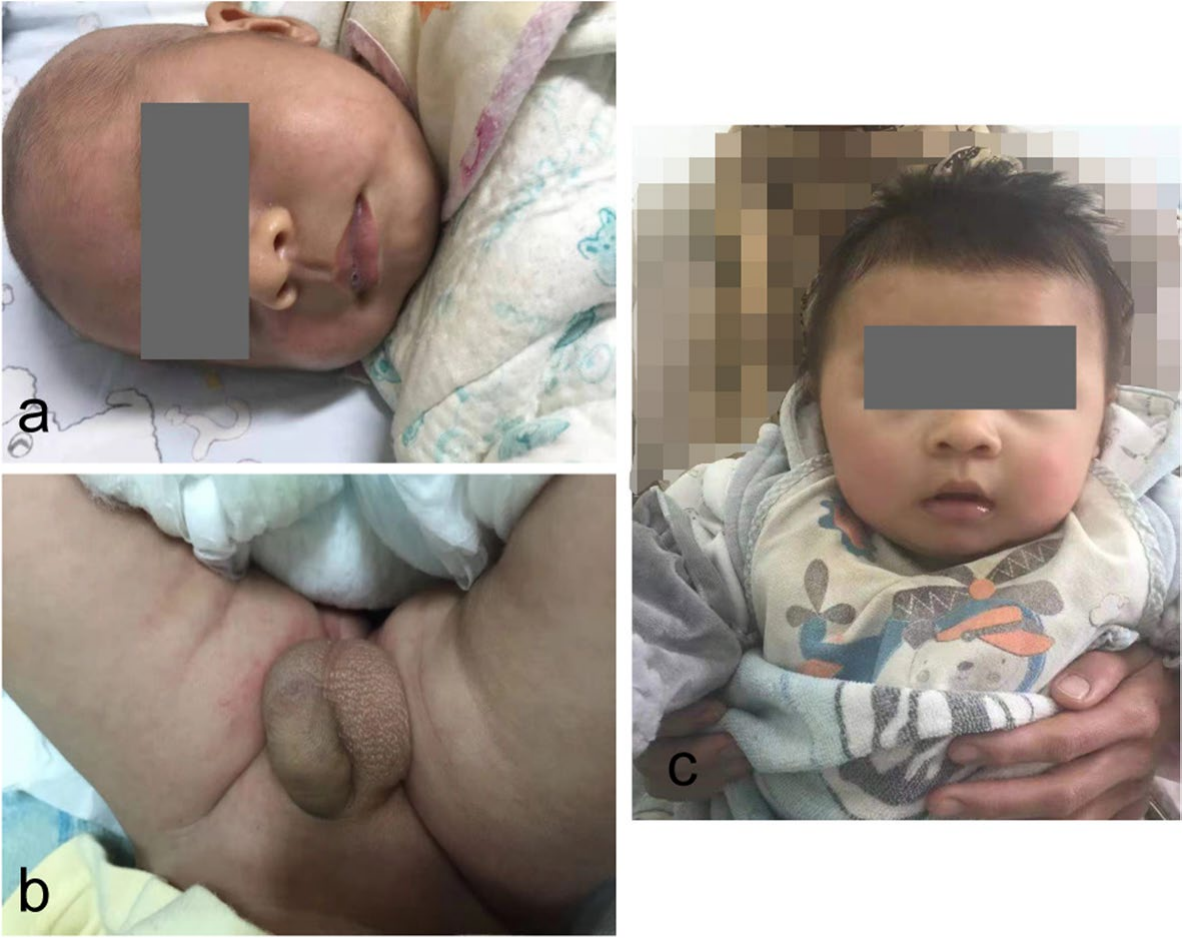
**a** and **b** show the appearance of this boy when admitting to our hospital. His skin was hyperpigmented and hypotonic without subcutaneous fat. **c** reveals the boy gained weight and his skin color was relatively normal after treatment at 5 months of age and his mother held him on her leg (the photo was cropped)

A 48-day-old full-term male baby was admitted to our hospital with “growth retardation and persistent vomiting for more than one month”. The parents were nonconsanguineous, the pregnancy and delivery periods were unremarkable. After birth, the child gradually developed symptoms including dark skin, failure to thrive, poor appetite, vomiting gastric contents without bile and coffee grounds, occasional diarrhea without mucus and blood, and was previously treated with “reduced glutathione”, “creatine phosphate”, “calcium gluconate”, “hydrocortisone”, and “metformin” in another hospital for 9 days before transferred to our tertiary hospital. During the treatment, he had no seizures, no screaming, no abdominal distension, and his condition did not alleviate. For further evaluation, his family was readmitted to our hospital. The birth weight of the male infant was 3300 g, but at the time of admission, his weight was 3500 (< 3rd percentile) and length was 61 cm (90th percentile) [7]. On physical examination, the infant was awake and dehydrated. The skin was hyperpigmented and less elastic without subcutaneous fat (Fig. 1a). His neck was supple. There was no facial dysmorphism noted. His lungs were clear, his heart rate was regular and no murmurs were found on auscultation. His abdomen was not tender, without organomegaly. Genital examination revealed penis scrotal hyperpigmentation and thickened penis, with normal testicles volume (1 ml on each side) (Fig. 1b). The neurologic examination was unremarkable. Routine laboratory tests revealed the following serum levels: potassium 5.9 mmol/L (normal range, 3.5 to 5.5), sodium 132 mmol/L (normal range, 135 to 145), alanine transaminases 141.1 U/L ( ALT, normal range: 0 to 40), aspartate transaminases 88.8 U/L (AST, normal range, 0 to 40), alkaline phosphatase 50.7 U/L (ALP, normal range, 147.7 to 309.3), creatine kinase 1586 U/L (CK, normal range, 16.5 to 211.5), creatine kinase isoenzyme 168 U/L (CK-MB, normal range, 16.5 to 211.5), α-hydroxybutyrate 314 U/L (α-HBDH, normal range, 75.5–211.5), lactate dehydrogenase isoenzyme 76U/L (LDH-MB, normal range, 30 to 120), lactate dehydrogenase 413U/L (LDH, normal range: 67 to 394.1). No clinically significant findings were noted on chest and abdomen radiography. An ultrasound cardiography revealed no other problems except for Patent Foramen Ovale. Abdominal and urinary ultrasound showed hepatomegaly and bilateral renal enlargement. Based on hyperkalemia, hyponatremia, penis scrotal hyperpigmentation, and his enlarged kidneys and liver, hydrocortisone (5 mg given intravenously every 8 h) and fludrocortisone (0.1 mg administered orally per day) were started or a presumed diagnosis of adrenal cortical hyperplasia (ACH). Glutathione and sodium fructose diphosphate were initiated orally for abnormal liver function and myocardial abnormalities especially. Over the next 3 weeks, the child’s appetite improved and the symptoms of dehydration were reduced. He increased by 0.5 kg in weight. Routine laboratories were re-ordered. The sodium level was 130 mmol/L (normal range, 135 to 145), potassium level 5.1 mmol/L (normal range, 3.5 to 5.5), ALT level 62.8 U/L (normal range, 0 to 40), AST level 53.3 U/L (normal range, 0 to 40), ALP level 147.2 U/L (normal range, 147.7 to 309.3), CK level 621 U/L (normal range, 16.5 to 211.5), CK-MB level 97 U/L (normal range, 15 to 80), LDH level 367.4 U/L (normal range, 67 to 394.1), LDH-MB level 64.9 U/L (normal range, 30 to 120), α-HBDH level 255.2 U/L (normal range, 75.5 to 211.5), 17-alpha hydroxyprogesterone 0.48 ng/L (17-α-OHP, normal range, 0 to 1.54). No lipid metabolism test was performed during the hospitalization. An adrenal function test showed no significant abnormalities (Table 1), and the results also contradicted the diagnosis of ACH. We revised the main diagnosis to probable adrenocortical insufficiency. The patient was discharged on day 23 after admission and continued oral treatment with reduced doses of cortisone acetate for relived symptoms, fludrocortisone, and sodium supplementation (Table 1).

**Table 1:** Medical history timeline during the first 1.8 years of life and biochemical and hormonal values

Chronologic Age(years)	40 + 3 weeks of GA (Birth)	46 + 6 weeks of GA (First hospitalization)	0.17(First discharge)	0.22 (Second hospitalization)	0.27 (Second discharge)	0.39	0.65	0.94	1.36	1.75 (Death)
Length(cm)	/	61	/	64	/	64.6	68	70	75	76
Weight(Kg)	3.3	3.5	/	4.3	4.8	6.2	8	8.3	9.6	10.4
Head Circumference(cm)	/	/	/	/	/	/	/	/	/	/
ACTH(7.2–63.6 pg/ml)	/	/	15.04	8.76	72.83	4.48	3.42	3.86	2.36	/
Cortisol(66–630 nmol/L)	/	/	647.9	874.4	12.72	29.88	11.57	6.41	7.64	/
LH(1.5–12.4 IU/L)	/	/	/	7.23	/	/	/	1.76	0.46	/
FSH(0.1–5.4 IU/L)	/	/	/	13.93	/	/	/	0.73	0.1	/
E2(18-73 pmol/L)	/	/	/	18.4	/	/	/	18.4	57.1	/
P(0.7–4.3 nmol/L)	/	/	/	0.1	/	/	/	0.1	0.65	/
T(0.1–1.12 nmol/L)	/	/	/	13.03	/	/	/	0.09	0.09	/
PRL(106–713 mIU/L)	/	/	/	518.3	/	/	/	131.6	243.6	/
17-α-OHP(0–1.54 ng/ml)	/	/	0.48	0.52	/	/	/	/	/	/
ALT(0–40 U/L)	/	141.1	62.8	115	138	233	153	181	183	439
AST(0–40 U/L)	/	88.8	53.3	121	173	253	163	206	212	1078
ALP(147.7–309.3 U/L)	/	50.7	147.2	247	252	210	149	155	123	140.8
CK(16.5–211.5 U/L)	/	1584	621	2620	6771	11,622	10,680	13,512	9822	26,055.3
CK-MB(15–80 U/L)	/	203.1	97	254	343	540	520	538	549	2353
LDH(67–394.1 U/L)	/	402.3	367.4	/	617	959	815	956	946	2440.4
LDH-MB(30–120 U/L)	/	125.4	64.9	/	88	116	97	101	110	165.2
TCHOL(3.12–5.2 mmol/L)	/	/	/	4.86	5.18	4.76	4.28	4.46	4.53	/
α-HBDH(75.5–211.5 U/L)	/	314	255.2	300	447	631	545	624	617	1560.9
TG(0.8–1.8 mmol/L)	/	/	/	12.01	8.87	4.76	6.71	6.09	4.53	/
Apo-A(1–1.6 g/L)	/	/	/	1.67	1.45	1.68	1.43	1.65	1.74	/
Apo-B(0.6–1.1 g/L)	/	/	/	0.6	0.8	0.67	0.6	0.49	0.61	/
Na(135–145 mmol/L)	/	133	130.4	132	136	139	142	141	143	140.8
K (3.5–5.5 mmol/L)	/	4.6	5.1	6.7	6.6	5	4.4	3.7	4.2	5.7
Ca(2.2–2.7 mmol/L)	/	2.39	2.33	2.86	2.68	2.62	2.53	2.34	2.32	1.97
Mg(0.6–0.95 mmol/L)	/	0.74	0.7	0.81	0.89	0.88	0.79	0.78	0.9	1.26
Cl(96–106 mmol/L)	/	110.7	97.6	94	96	101	102	103	101	98
Phosphorus(1.45–2.1 mmol/L)	/	1.08	1.54	2.11	2.45	2.16	1.84	1.76	1.78	3.48
Lac(0.5–2.2 mmol/L)	/	1	2.5	4.2	/	/	/	/	/	10.1
Glu(3.9–5.8 mmol/L)	/	3.27	4.5	4.1	5.4	/	3.6	/	/	1.7
Medication		given hydrocortisone acetate intravenously, 5 mg, q8h + oral fludrocortisone, 0.1 mg/d	oral hydrocortisone acetate, 5 mg in the morning, 2.5 mg at noon and 2.5 mg at night + oral fludrocortisone, 0.1 mg/d + salt supplementation 2 g/day	oral hydrocortisone acetate, 5 mg- 2.5 mg- 2.5 mg + oral fludrocortisone, 0.1 mg/d + salt supplementation 2 g/day	same as the previous	oral hydrocortisone acetate, 2 mg- 2 mg- 2 mg + oral fludrocortisone, 0.1 mg/d + salt supplementation 2 g/day	same as the previous	same as the previous	same as the previous	/

*GA* Gestational age, *ACTH* Adrenocorticotropic hormone, *LH* Luteinizing hormone, *FSH* Follicle stimulating hormone, *E2* Estradiol, *P* Progesterone, *T* Testosterone, *PRL* Prolactin, *17-α-OHP* 17-alpha hydroxyprogesterone, *ALT* Alanine transaminases, *AST* Aspartate transaminases, *ALP* Alkaline phosphatase, *CK* Creatine kinase, *CK-MB* Creatine kinase isoenzyme, *LDH* Lactate dehydrogenase, *LDH-MB* Lactate dehydrogenase isoenzyme, *TCHOL* Total cholesterol, *α-HBDH* α-hydroxybutyrate, *TG* Triglycerides, *Na* Sodium, *K* Potassium, *Ca* Calcium, *Mg* Magnesium, *Cl* Chlorine, *Lac* Lactic acid, *Glu* Glucose

**Fig. 2:**
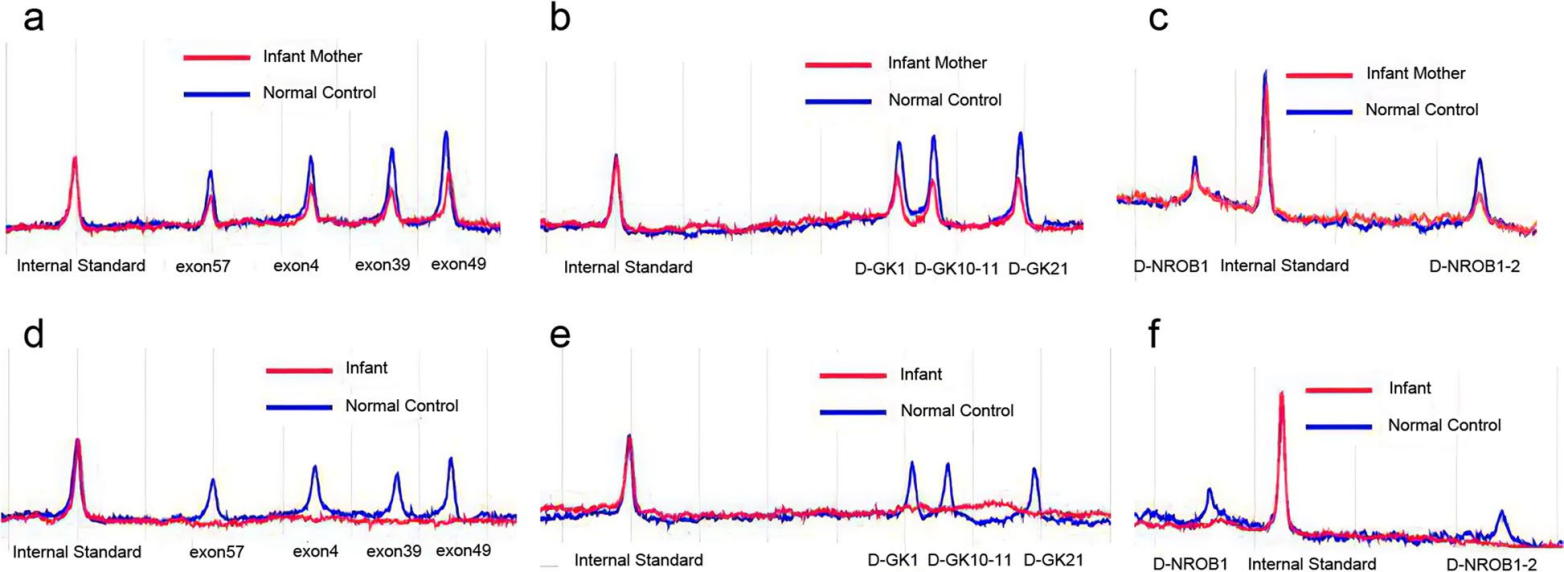
Results of gene screening via multiplex PCR amplification and DHPLC analysis. **a**, **b**, and **c** demonstrate all exons of the DMD, GK, and NROB1 genes were heterozygous deletions in samples of this boy’s mother. While **d**, **e**, and **f** reveal deletions of DMD, GK, and NROB1 genes of this boy

At a follow-up visit one week after discharge, his weight increased to 4.3 kg but an outpatient electrolyte test revealed an elevated serum level of potassium (6.7 mmol/L) and decreased serum level of sodium (132 mmol/L). The child was admitted again because the cause of the elevated serum potassium was unclear. On examination, his response was poor, while vital signs were still stable. His skin hyperpigmentation and genital enlargement persisted. A lipid metabolism analysis after admission revealed Apo-A 1.67 g/L (normal range, 1 to 1.6), Apo-B 0.6 g/L (normal range, 0.6 to 1.1), total cholesterol 4.51 mmol/L (TCHOL, normal range, 3.12 to 5.2), and triglycerides 12.01 mmol/L (TG, normal range, 0.8 to 1.8). The value of 17-α-OHP was still within normal limits (Table 1), compared to that of the previous admission. A following urine gas chromatography-mass spectrometry (GC–MS) analysis showed that the urine was positive for glycerol, which reached 3129.2 umol/mmol (normally negative). Due to positive urinary glycerol and triglyceridemia, clinical suspicion was raised for GKD. He continued to be treated with steroid replacement and sodium chloride supplementation (10% sodium chloride 20 ml given intravenously per day). After obtained informed consent from the child’s parents, His and his mother's peripheral blood samples were sent to the molecular pathology center for free gene screening via multiplex PCR amplification and DHPLC analysis. In his mother’s samples, all exons of the DMD, GK, and NROB1 genes were heterozygous deletions (Fig. 2a, b, c), while deletions of DMD, GK, and NROB1 genes in the boy’s samples were confirmed (Fig. 2d, e, f). All of these genes were located in the Xp212region. Eventually, GKD was confirmed in the male infant. After his symptoms of dehydration diminished, the baby boy was discharged again on day 20 after hospitalization with oral cortisone acetate, fludrocortisone, and salt supplementation (Table 1).

At follow-up visits at 5, 8, and 12 months of age, the infant continued to gain weight and length. His skin’s dark color gradually faded (Fig. 1c) and the dose of hydrocortisone acetate had been reduced to 2 mg every 8 h as the patient’s symptoms remained stable (Table 1). At his 8-month follow-up visit, an ultrasound revealed normal liver and kidney size, but mild splenomegaly. And at his follow-up visit four months later, abdominal and urinary ultrasound revealed no abnormalities. He could not sit alone until he was 1 year 4 months old, and he could not call his mommy and daddy yet, only babbling. Unfortunately, the last time we saw him he was very sick when he was presented to the emergency department gasping for 1 week. His parents told us that they neglected to take him to the pediatrician in time when he developed symptoms of runny nose, cough, vomiting, and poor appetite. Based on the pre-admission symptoms, it can be inferred that he had a previous upper respiratory tract infection. On admission, his respiratory rate was 35 to 50 bpm, heart rate was 120 bpm, blood pressure was 60/40 mmHg, and SpO_2_ was 69% checked by a finger oximeter. He was afebrile, poor response. His skin was clammy and ashen with cyanosis in the lips. His chest had wet rales to auscultation. His breathing and heartbeat rhythms were irregular. His abdomen was soft without organomegaly or mass. His extremities were cold, and CRT (capillary refill time) was greater than 5 s. His muscle strength revealed hypotonic. We immediately began resuscitation, opened intravenous access, and supplemented saline with 20 ml/kg to expand the blood volume. He was placed on high-flow oxygen via a mask immediately. Saline with 20 ml/kg was supplemented to expand his blood volume via the intravenous route. 50 mg of hydrocortisone was administered intravenously to maintain adrenal cortical function simultaneously. Peripheral blood gas revealed hypoglycemia, hyperkalemia, hyperlactatemia (Table 1), and hypoxemia (PaO_2_ 25.3 mmHg, normal range, 80 to 100; PaCO_2_ 58 mmHg, normal range, 35 to 45). The blood gas analysis also showed severe mixed acidosis (pH 7.166, normal range, 7.35 to 7.45; BE -7.7 mmol/L, normal range, -3 to 3; HCO_3_^−^std 17.4 mmol/L, normal range, 22 to 27; HCO_3_^−^ 20.9 mmol/L, normal range, 22 to 27; SpO_2_ 22.8%, normal range, 95 to 98; FiO_2_ 54%). After 30 min of resuscitation, there was no significant improvement in his symptoms. His breathing rate dropped to 10 to 15 bpm, and he was soon intubated. However, he went into cardiac arrest, and the family gave up resuscitation. The child, at 1 year 9 months old, died of severe pneumonia combined with type 2 respiratory failure and hypoglycemia after timely resuscitation.

## Discussion and conclusions

Complex glycerol kinase deficiency is due to deletions of closely linked loci on the Xp21 chromosome and is often hard to diagnose in its early stage due to the low prevalence and the lack of awareness among physicians. The main affected people are usually male, and so far, not more than 10 cases of female patients have been reported [5, 8–10]. Xp21 contiguous gene deletion syndrome is classified into three types in terms of clinical presentation [3]. Infantile or complex GKD is the most common type, caused by deletions of GKD, and its neighboring genes DMD and AHC (NR0B1). Juvenile GKD and Adult GKD both result from point mutations in GK. The former is symptomatic, with symptoms such as acidemia, episodic vomiting, and coma. The latter is usually asymptomatic and benign, detected incidentally due to pseudohypertriglyceridemia [11]. Generally, the infantile type has the most complex clinical presentation, which is difficult to diagnose and is the most in need of early diagnosis and treatment.

In this case, the patient presented with vomiting, nausea, failure to gain weight, and skin hyperpigmentation, which easily led to the diagnosis of congenital adrenal hyperplasia [12]. Follow-up investigations revealed normal serum levels of ACTH and 17-α-OPH, and the diagnosis was revised to adrenocortical insufficiency. The normal ACTH serum level in the child may be due to early steroid replacement therapy. During the first hospitalization, Investigations of lipid metabolism were overlooked, which led us to lose the opportunity to suspect CGKD. Deletion of the GK locus results in glycerol kinase deficiency associated with hypertriglyceridemia. If elevated triglyceride levels are found on lipid metabolism tests in an infant with growth retardation, then the diagnosis of CGKD should be suspected. To date, a fast and simple way to diagnose CGKD is to determine the urine glycerol level via GC–MS analysis [4]. Genetic screening then could confirm deletions of CGKD loci.

Our patient had deletions of DMD, GK, and NROB1(AHC) genes. Like other similar patients, his symptoms of AHC appeared earlier, such as hyperkalemia, hyponatremia, poor appetite, and growth retardation, which were also the main reasons for clinic visits. Clinicians usually consider congenital adrenal hyperplasia when these symptoms occur because the incidence of congenital adrenal hyperplasia is 1:5 000 to 1:20 000 [13], which is significantly higher than the 1:140 000 to 1:12 000 000 incidence [14] of AHC. Therefore, lipid metabolism testing is essential in the early differential diagnosis when an infant presenting with symptoms of adrenal insufficiency.

While the symptoms of DMD in infants are usually difficult to observe, parents may only pay attention to hypotonia when the child is still unable to sit until 6 months of age or older. Our patient presented motor and mental retardation after 1 year of age. He cannot sit alone and call his mom and dad. In clinic practice, we can infer the possibility of DMD in terms of laboratory findings. The serum level of CK raises markedly at least 10 to 20 times higher than the upper limit of normal [15]. In our patient, the serum CK level was about 7.5 times the upper limit of normal. After corticosteroid therapy, the serum CK level did not decrease. We considered that one of the reasons was that the patient’s extensive muscles were also involved. In addition, male patients with DMD often accompany mental retardation [16], and this child also presented with delayed speech development after 1 year of age.

In general, corticosteroid replacement and sodium supplementation are conventional therapies for CGKD [2, 17]. Steroid replacement therapy could ensure the growth of the affected child. The use of fludrocortisone prevents sodium loss and helps to correct hyponatremia and hyperkalemia either. Hydrocortisone acetate and fludrocortisone were initiated after his admission. The serum TG level gradually dropped to the normal range after treatment. Salt supplementation was also administrated when adrenal insufficiency was considered. Eventually, the child responded well to corticosteroid replacement therapy and continued to thrive. The ACTH and cortisol levels, however, decreased and remained at a lower level, especially the latter, after corticosteroid replacement therapy started for one month. We prescribed a slightly larger loading dose of corticosteroids, which is a common practice when treating infants, to downregulate metabolic levels and end-organ resistance [18]. Additionally, the HPA axis may have been suppressed because of the higher amounts of corticosteroids we prescribed to the child. Also, his immune system may have been inhibited and he developed acute adrenal crisis after the upper respiratory infection, which led to the internal environment disturbance with respiratory failure, hyperkalemia, and hypoglycemia.

In summary, the case highlights AHC cannot be ruled out in infants with persistent vomiting as the main symptom after excluding gastrointestinal disorders. CGKD is difficult to diagnose and a thorough routine examination can help detect pseudohypertriglyceridemia and elevated CK for further gene screening. The dose of corticosteroid replacement therapy for CGKD needs to be adjusted dynamically to avoid aggravating the effect on the HPA axis and immune system of the child and to avoid adrenal crisis as much as possible.

## Data Availability

Not applicable.

## Abbreviations

AHC: Adrenal hypoplasia
DMD: Duchenne muscular dystrophy
CGD: Chronic granulomatosis
CGKD: Complex glycerol kinase deficiency
ACH: Adrenal cortical hyperplasia
ACTH: Adrenocorticotropic hormone
GK: Glycerol kinase
OTC: Ornithine carbamoyltransferase
GKD: Glycerol kinase deficiency
GA: Gestational age
LH: Luteinizing hormone
FSH: Follicle-stimulating hormone
E2: Estradiol
P: Progesterone
T: Testosterone
PRL: Prolactin
17-α-OHP: 17-Alpha hydroxyprogesterone
ALT: Alanine transaminases
AST: Aspartate transaminases
ALP: Alkaline phosphatase
CK: Creatine kinase
CK-MB: Creatine kinase isoenzyme
LDH: Lactate dehydrogenase
LDH-MB: Lactate dehydrogenase isoenzyme
TCHOL: Total cholesterol
α-HBDH: α-Hydroxybutyrate
TG: Triglycerides
PCR: Polymerase chain reaction
DHPLC: Denaturing high-performance liquid chromatography
Na: Sodium
K: Potassium
Ca: Calcium
Mg: Magnesium
Cl: Chlorine
Lac: Lactic acid
Glu: Glucose
CRT: Capillary refill time
SpO_2_: Oxygen saturation
PaO_2_: Partial pressure of oxygen
PaCO_2_: Partial pressure of carbon dioxide
HCO_3_^−^: Bicarbonate ion
HCO_3_^−^std: Standard bicarbonate ion
FiO_2_: Inhale oxygen flow
